# High-Performance Porous Ceramics from Red Mud via Foam-Gelcasting for Efficient Waste Utilization

**DOI:** 10.3390/ma19091817

**Published:** 2026-04-29

**Authors:** Yanxiu Wang, Fan Wang, Ling Zhang, Shipei Wang, Anqi Yang, Chengwen Wang, Li Wang, Haisheng Han, Wei Sun

**Affiliations:** 1 School of Minerals Processing and Bioengineering, Central South University, Changsha 410083, China; 222066@csu.edu.cn (Y.W.);; 2 Key Laboratory of Hunan Province for Clean and Efficient Utilization of Strategic Calcium-Containing Mineral Resources, Changsha 410083, China; 3 College of Engineering, Mathematics and Physical Sciences, University of Exeter, Exeter EX4 4QF, UK

**Keywords:** red mud, porous ceramic, foam-gelcasting, thermodynamic calculation, phase evolution, waste disposal

## Abstract

Red mud, as a by-product of alkaline regeneration of alumina, has limited application due to its strong alkalinity, fine particle size, and complex composition. In this work, red mud porous ceramics with uniform pore size distribution and high mechanical strength were prepared using a foam-gel casting method. The effects of solid loading and sintering temperature on the microstructure of porous ceramics were systematically investigated. The porosity of red mud-based porousceramics sintered at 1150 °C with a solid content of 60.4% was 33.7%, and the maximum compressive strength was 54.70 MPa, while the porousceramics prepared with a solid loading of 34.1% and sintered at 1050 °C achieved a maximum porosity of 79.7% and a compressive strength of 2.36 MPa. Increasing the solid loading reduced porosity and enhanced compressive strength, allowing for the tailoring of mechanical properties to meet specific application requirements. Higher sintering temperature promoted the formation of the liquid phase, enhanced particle bonding, and further improved the compressive strength. Additionally, toxicity leaching tests confirmed that the ceramics are environmentally safe, with leachate levels well within regulated limits. These results demonstrate the potential of foam-gel casting as an effective route for transforming red mud into value-added porous ceramics, thereby contributing to sustainable waste utilization and broadening the application prospects of red mud-based materials.

## 1. Introduction

Red mud is unavoidable solid waste generated from the Bayer process, which refines bauxite and produces aluminum oxide. In general, the fabrication of 1 ton of aluminum will produce 1–1.5 tons of red mud (RM), and 120 million tons of RM have been produced annually worldwide [1,2,3,4,5]. The difficulty in utilizing red mud is mainly due to the elevated pH (about 10~13), large quantity, and ultra-refined particle size [6]. The accumulation of large quantities of red mud is very hazardous and toxic for the environment. Therefore, recycling red mud is a crucial and urgent challenge.

To minimize the waste of resources and the impact on the environment, researchers worldwide have made significant efforts in the reuse of red mud, such as the extraction of valuable metals [7,8,9], creation of construction materials [10,11,12,13,14,15], employment as catalysts [16], waste water and waste gas treatment [17,18], and improvement of soil [19,20]. Red mud contains a great deal of valuable elements (Si, Al, Ca, etc.) that can be used in the preparation of ceramics, and it contains a lot of alkali metal oxides, which are excellent fluxes for lowering the sintering temperature of ceramics. In addition, an elevated sintering temperature can solidify some alkali and other heavy metal ions within the ceramic matrix, thereby reducing their leaching risk and associated environmental impact [21,22,23]. Therefore, the use of red mud as a sintering aid in the preparation of ceramics is a good strategy for reusing red mud. Meanwhile, the preparation of porous ceramics from solid waste has attracted great attention since it is feasible to efficiently convert solid waste in large quantities into porous ceramics with numerous features (lightweight, low thermal conductivity, high strength, etc.), and thus to realize high added-value utilization. Currently, the preparation of porous ceramics from red mud is almost exclusively done by the high-temperature chemical blowing method [24,25,26,27,28,29,30,31], and the foaming agents used include CaCO_3_, MgCO_3_, Na_2_SiO_3_, graphite, SiC, Na_3_PO_4_ etc. However, high-temperature chemical blowing has several drawbacks, including uneven pore size distribution, uncontrolled pore morphology, and relatively large pores. Foam-gel casting is a well-recognized method for the fabrication of porous ceramics [32,33,34,35,36] owing to its simplicity, cost efficiency, and high yield. However, it has not been applied to the preparation of red mud-based porous ceramics. Compared with previously reported red mud-based porous ceramics prepared mainly by high-temperature chemical blowing, the present work introduces foam-gel casting as an alternative route for producing red mud-based porous ceramics with a more uniform pore structure and tunable mechanical properties.

In this study, red mud porous ceramics with uniform pore size distribution and high strength were prepared directly by the foam-gel casting method. Compared with previously reported red mud-based porous ceramics, which are mainly fabricated by high-temperature chemical blowing, the present work introduces foam-gel casting as an alternative route for producing red mud-based porous ceramics with a more uniform pore structure and tunable mechanical performance. In addition, the combination of raw material characterization, thermodynamic analysis, and leaching safety evaluation further highlights the novelty of this study. The objectives of this study were to characterize the red mud raw material in terms of its chemical composition, phase composition, morphology, particle size distribution, and liquidus temperature; to investigate the effects of solid loading and sintering temperature on the microstructure, porosity, density, compressive strength, and phase composition of the prepared porous ceramics; and to evaluate the environmental safety of the sintered products through leaching tests. This study provides an effective approach for the large-scale utilization of red mud and for mitigating its environmental impact.

## 2. Materials and Methods

### 2.1. Raw Materials

The raw material of red mud used in this experiment originated from the Bayer process at the Shandong Alumina Plant in China. The red mud’s chemical composition was measured using X-ray fluorescence spectrometry (XRF), and the results are displayed in Table 1. Cetyltrimethylammonium bromide (CTAB), acrylate copolymer (AC, (C_3_H_4_O_2_)_n_), and carboxymethyl cellulose sodium (CMC) were purchased from Sinopharm Chemical Reagent Co., Ltd., Shanghai, China. CTAB served as both a dispersant and a foaming agent, while AC and CMC functioned as gelling agents. The plant-based protein foaming agent used in this study was purchased from Xi’an Yunyue Biotechnology Co., Xi’an, China; its main ingredient is triterpene saponin. Prior to preparing the porous ceramic slurry, CMC, CTAB, AC and PP were configured as 1.5 wt. %, 2.5 wt. %, 2.5 wt. % and 2.5 wt. % aqueous solutions, respectively.

### 2.2. Preparation of Red Mud-Based Porous Ceramics

The initial step involved mixing 60 g of red mud powder with 66.5 g of water, 24 mL of CMC, and 12 mL of PP, followed by 10 min of mechanical stirring to ensure thorough blending. In the subsequent step, 12 mL of CTAB and 1.5 mL of AC were introduced into the slurry with constant agitation. The mixture continued to be mechanically stirred for 10 min to induce stable foaming. The foamed slurry was then placed in a 4.5 × 4.5 × 2.5 cm rubber mold. After the slurry was poured into the mold, it was quickly put into the electric oven, dried at 35 °C for 12 h, and then dried at 80 °C for 8 h to make the green blank completely dry. The green bodies were sintered in a muffle furnace by heating at 1 °C/min to 400 °C and holding for 0.5 h to ensure the gradual burnout of residual moisture and organic additives, thereby avoiding cracking or collapse of the porous structure. The temperature then increased at 3 °C/min to the target temperature and held for 0.5 h before natural cooling to room temperature. The influence of the solid loadings of red mud (34.1–60.4 wt. %) and sintering temperatures (1050–1150 °C) on the microstructure of the fabricated porous ceramics was studied. The contents of CTAB, AC, and CMC were kept constant in all experiments; therefore, they were not treated as independent variables in the analyses of density, porosity, or pore structure.

### 2.3. Characterization Techniques

The particle size distribution of the red mud powder was analyzed using a laser particle size analyzer (Mastersizer 2000 with Hydro2000M, Malvern Panalytical, Worcestershire, UK). XRD analysis (D8 Advance, Bruker, Karlsruhe, Germany) was performed to identify the phase compositions of the raw material and the sintered porous ceramics, with a scanning range from 10° to 80° (2θ) at a speed of 2°/min under Cu Kα radiation. The morphology of the red mud powder and the microstructure of the sintered porous ceramics were observed using field-emission scanning electron microscopy (FE-SEM, JSM-IT500LV, Tokyo, Japan). To identify the equilibrium phase composition across a range of temperatures, calculations were performed with the equilibrium model in FactSage 8.0 software. For thermal analysis, the raw material underwent TG-DSC testing on a NETZSCH STA 449 F3 Jupiter thermal analyzer (NETZSCH STA 449 F3 Jupiter, NETZSCH, Selb, Germany), heated to 1200 °C in air at a rate of 10 °C/min. The porosity (*P*) of the sintered ceramics was then evaluated based on the following equation:

(1) $$P=\left(1-\frac{\rho }{\rho_{0}}\right)\times 100\%=\left(1-\frac{m/V}{\rho_{0}}\right)\times 100\%$$

where *m* is the mass, *V* is the volume, ρ is the bulk density of the porous sample, and $\rho_{0}$ is the true density of the sample.

The determination of true density was carried out with a Micromeritics AccuPyc 1330 analyzer (AccuPyc 1330, Micromeritics, Norcross, GA, USA). Mechanical performance under compression was characterized by the as-prepared porous ceramics using a WDW-QT50 universal testing machine (WDW-QT50, Jinan Shijin Group, Jinan, China), employing a constant displacement rate of 0.5 mm/min.

## 3. Results and Discussion

### 3.1. Characterization of Red Mud

**Figure 1:**
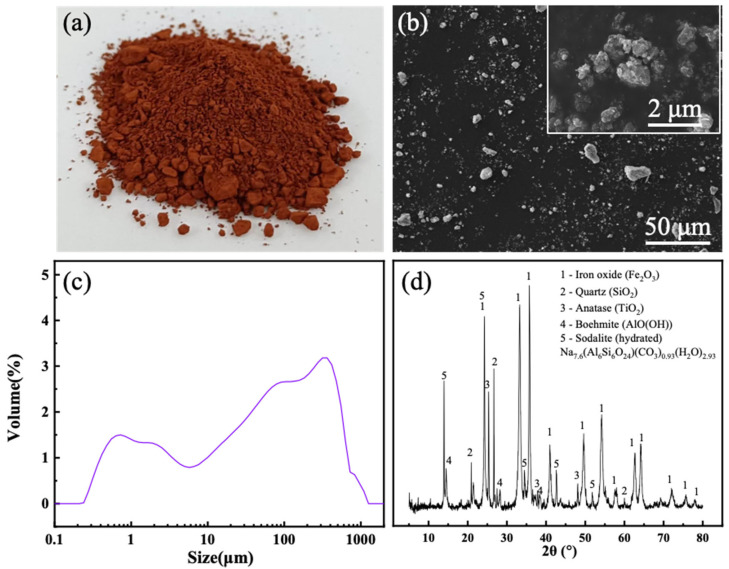
Characterization of the red mud powder. (**a**) macroscopic appearance, (**b**) SEM micrograph, (**c**) particle size distribution, and (**d**) XRD pattern.

Figure 1a–d show the macroscopic morphology, microscopic morphology, XRD pattern, and particle size distribution of red mud powder, respectively. Red mud powder contains a portion of large particles, with small particles generally below 20 µm in size (Figure 1b). As shown in Figure 1c, a portion of the particles is around 1 µm in size, and a portion of the particles is several hundred µm in size. The particle sizes of the red mud were mainly distributed in the range of 0.3–1660 μm, of which D_50_ is 60.9 μm. The crystalline phases of the red mud included the iron oxide (Fe_2_O_3_, 01-079-0007), quartz (SiO_2_, 01-085-0795), anatase (TiO_2_, 01-083-2243), boehmite (AlO(OH), 01-083-2384) and hydrated sodalite (Na_7.6_(Al_6_Si_6_O_24_)(CO_3_)_0.93_(H_2_O)_2.93_, 01-089-9099) phases.

**Figure 2:**
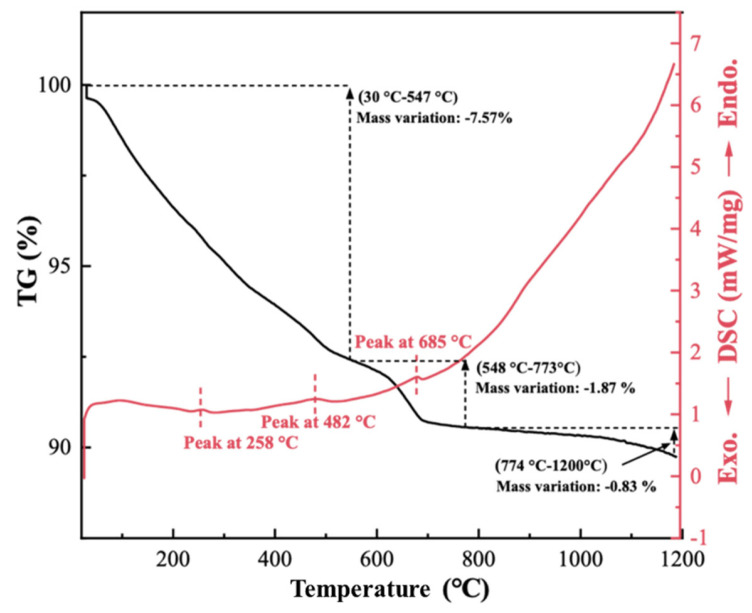
TG-DSC curve of red mud.

**Figure 3:**
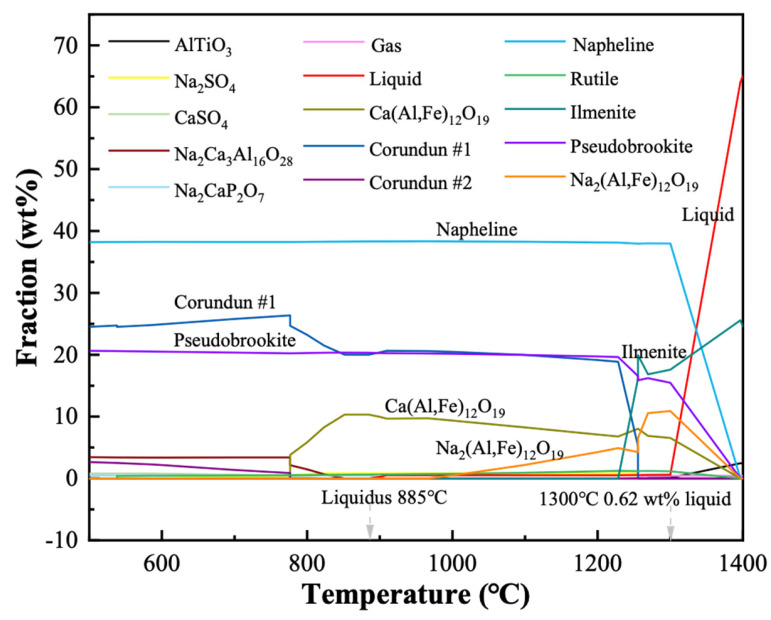
FactSage calculation of phase composition of red mud using equilibrium model.

In order to understand the thermal behavior of red mud and to determine the sintering temperature, TG-DSC analysis and FactSage calculations were carried out, and the results are shown in Figure 2 and Figure 3, respectively. According to the TG curve, there was a total mass loss of 10.27% for red mud up to 1200 °C. The first mass loss (7.57%) occurred in the temperature range 30–547 °C, which may be related to the evaporation of free water, bonded water in hydrated sodalite, and the decomposition of Al-bearing hydroxylated phases [37,38]. The thermal events observed in this range may also involve trace or poorly crystalline components that were not clearly resolved by XRD. The mass loss between 548 °C and 773 °C is attributed to the decomposition of minor carbonate-containing phases [38,39].

The third stage occurred after 773 °C, where the mass loss of the red mud was very small (0.83%), while the changing slope of the DSC curve may be ascribed to the beginning of the sintering process. The liquidus temperature of the red mud was calculated by the FactSage software to be 885 °C (Figure 3), at which point the mixture starts to produce a liquid phase. There is no obvious endothermic peak near 885 °C on the DSC curve, probably because the quantity of liquid phase formed is too limited. At 1300 °C, the amount of liquid phase reaches 0.62 wt. % and increases dramatically after 1300 °C. Excess liquid phase can cause the porous ceramic to collapse, and a small amount of liquid phase helps the particles to bond, so sintering temperatures below 1300 °C are optimal. Based on this, porous ceramics were chosen to be sintered at 1050, 1100, and 1150 °C in this work.

### 3.2. Effect of Solid Loading

**Figure 4:**
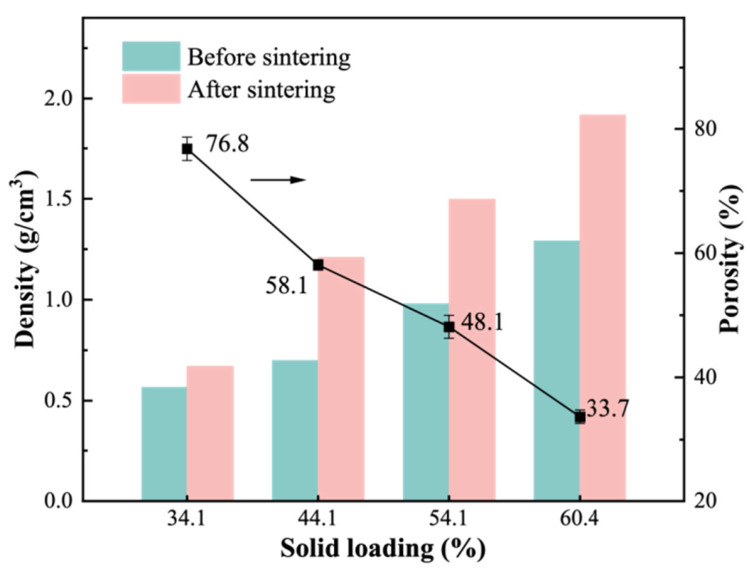
Density and porosity of the fabricated porous ceramics before and after sintering at 1150 °C as a function of solid loading.

Figure 4 illustrates the influence of different solid loadings on the density and porosity of samples before and after sintering at 1150 °C. The amounts of CTAB, AC, and CMC were kept constant for all samples; therefore, the variations shown in Figure 4 are attributed to the change in solid loading. With the solid loading increasing from 34.1% to 60.4%, the densities of both the green body and the fabricated ceramics steadily improved, and the porosity of the fabricated ceramics decreased significantly, from 76.8% to 33.7%. Solid loading has an important influence on porosity and density because an increase in solid loading results in an increase in the viscosity of the slurry, which can greatly reduce the foaming ability.

**Figure 5:**
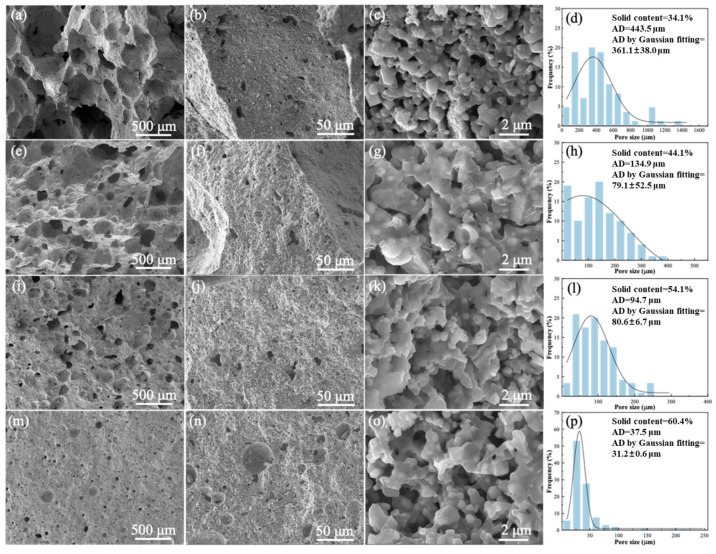
Microstructural characteristics of porous ceramics fabricated at varying solid loadings, accompanied by their respective pore size distribution histograms: (**a**–**d**) 34.1 wt. %; (**e**–**h**) 44.1 wt. %; (**i**–**l**) 54.1 wt. %; (**m**–**p**) 60.4 wt. %.

Figure 5 shows the microstructure of the red mud-based porous ceramics prepared with different solid loadings and their relevant pore size distribution plots. The samples prepared at a solid loading of 34.1% exhibited a structure of unevenly distributed macropores (100–1000 µm), with a few small pores on the inner walls of the cells. This is due to the higher water content at low solid loading, which facilitated air incorporation during mechanical stirring and contributed to the formation of large and unevenly distributed pores. At the same time, there was also enough water to maintain the stability of the gas–liquid interface in each bubble.

As the solid content increased, it is clear that the samples became denser, which was manifested by a decrease in the amount of pores, along with smaller pore size. When the solid loading increased, the water content of the slurry decreased, which reduced air incorporation during foaming and consequently led to smaller pores in the porous ceramics. Moreover, the amount of water at this time did not maintain the stability of the gas–liquid interface in a large number of large bubbles. As a result, the volume of the bubbles in the slurry became smaller, resulting in smaller pores in the porous ceramics.

**Figure 6:**
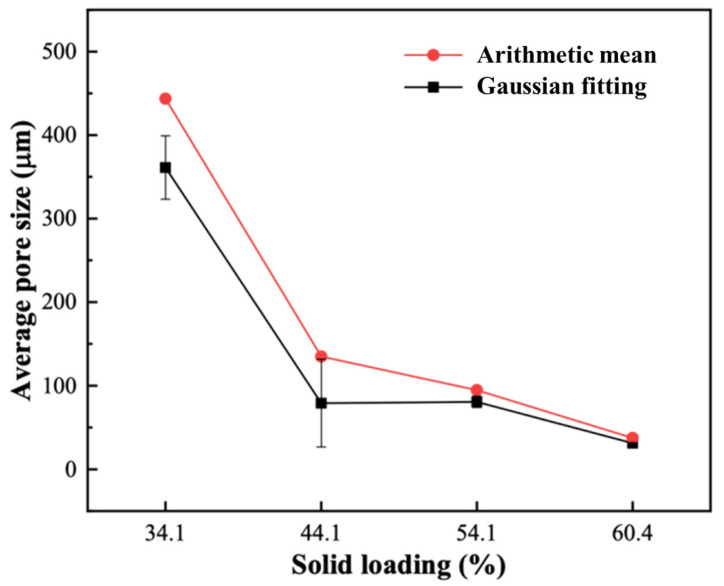
Average pore diameter as a function of solid loading.

High solid loading porous ceramics with smaller pores can be further confirmed from the pore size distribution plots (Figure 5d,h,l,p). For the porous ceramics prepared with a solid loading of 34.1 wt. %, the pore diameters mainly ranged from 60.8 to 1600 μm, with an average diameter (AD) of 443.5 μm and a Gaussian-fitted AD of 361.1 μm. When the solid loading increased to 44.1 wt. %, the pore size distribution became significantly narrower, which was mainly associated with the reduction of large pores and interconnected macropores, together with an increase in the fraction of small pores. With a further increase in solid loading to 54.1 wt. % and 60.4 wt. %, the pore size continued to decrease, mainly due to the continued suppression of large pores and the overall refinement of the pore structure, rather than solely because of an increase in the smallest pore-size fraction. The AD and Gaussian-fitted AD as a function of solid loading are presented in Figure 6, and both values gradually declined with increasing solid loading. It can therefore be concluded that increasing the solid loading significantly reduced the pore size and narrowed the pore size distribution.

### 3.3. Sintering Temperature

**Figure 7:**
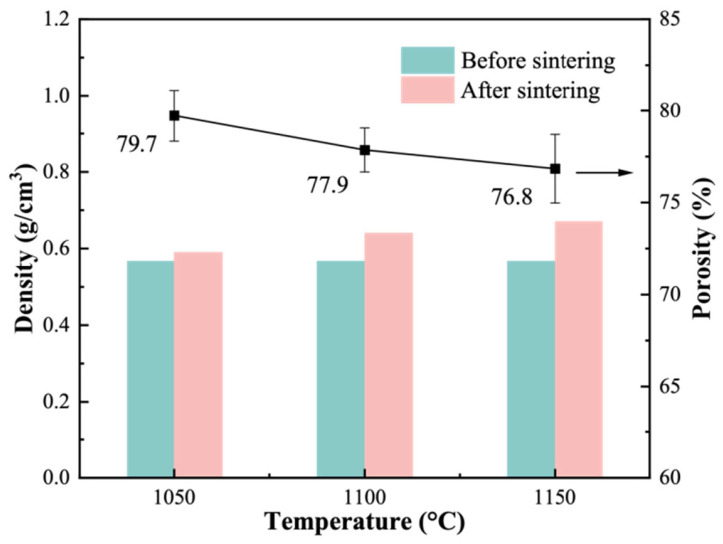
Variations in the density and porosity of the fabricated porous ceramics as a function of the sintering temperature.

**Figure 8:**
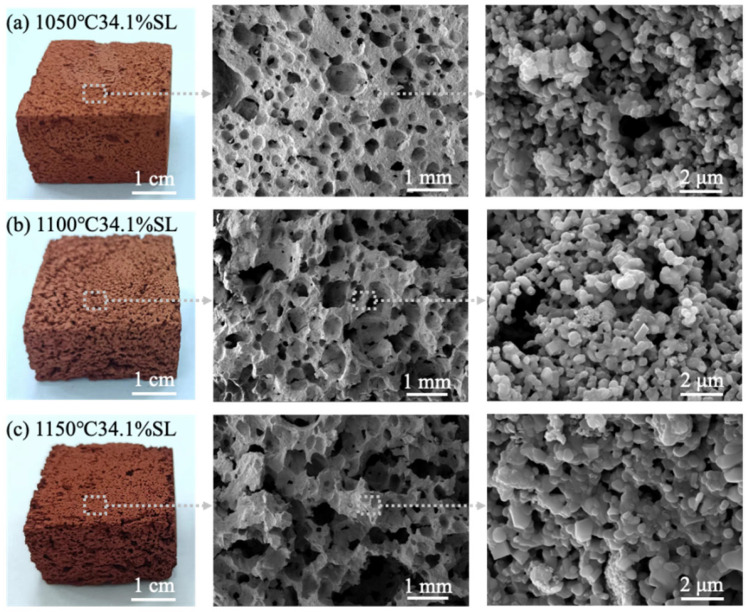
Evolution of the macroscopic and microscopic morphology of the fabricated porous ceramics prepared at different temperatures and a fixed solid loading of 34.1 wt. %: (**a**) 1050 °C; (**b**) 1100 °C; (**c**) 1150 °C.

In this section, the effect of sintering temperature was investigated using the sample with 34.1 wt. % solid loading as a representative high-porosity composition in order to evaluate the influence of thermal treatment on pore retention, densification, and compressive strength. Figure 7 presents the variations in density and porosity with sintering temperature for samples containing 34.1 wt. % solids. The contents of CTAB, AC, and CMC were unchanged; thus, the differences shown in Figure 7 and Figure 8 reflect the effect of sintering temperature under a fixed solid loading of 34.1 wt. %. An initial density of 0.57 g/cm^3^ was measured for the dried green bodies. Sintering from 1050 to 1150 °C led to a density increase to 0.67 g/cm^3^, accompanied by a reduction in porosity from 79.7% to 76.8%.

Photographs and microstructural images of the fabricated porous ceramics sintered at 1050, 1100, and 1150 °C are shown in Figure 8. Red mud porous ceramics remained red after high-temperature sintering. Although the overall porous structure was maintained at different sintering temperatures, some differences in pore morphology were observed. The sample sintered at 1050 °C exhibited a relatively more uniform pore structure with a higher proportion of smaller pores, whereas larger pores became more evident in the samples sintered at 1100 °C and especially at 1150 °C. From the enlarged SEM images, it can be observed that the red mud powder sintered and merged into large-sized dense grains with increasing temperature. Elevated temperatures facilitate increased liquid phase generation and a consequent enhancement in particle sintering ability, both factors contributing significantly to improved density and mechanical strength in the ceramic body.

**Figure 9:**
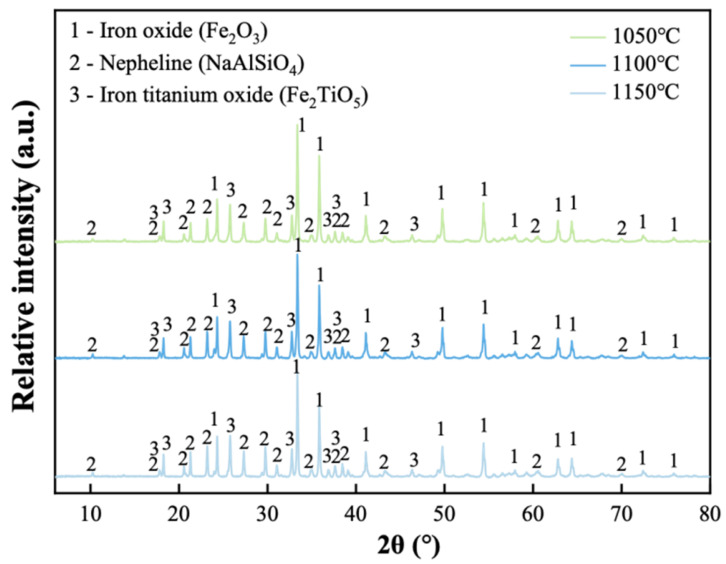
XRD patterns of porous ceramics sintered at different temperatures.

Figure 9 reveals the XRD patterns of porous ceramics sintered at different temperatures. The porous ceramics sintered at different temperatures all had similar XRD patterns, and the main phases were iron oxide (Fe_2_O_3_), nepheline, and iron titanium oxide (Fe_2_TiO_5_). A rise in temperature led to a slight decrease in the relative intensity of the iron oxide diffraction peaks, contrasting with an observed increase for nepheline. The experimental XRD results corroborate the FactSage thermodynamic predictions, indicating nepheline, iron oxide, and pseudobrookite as the major phases present.

### 3.4. Compressive Strength

**Figure 10:**
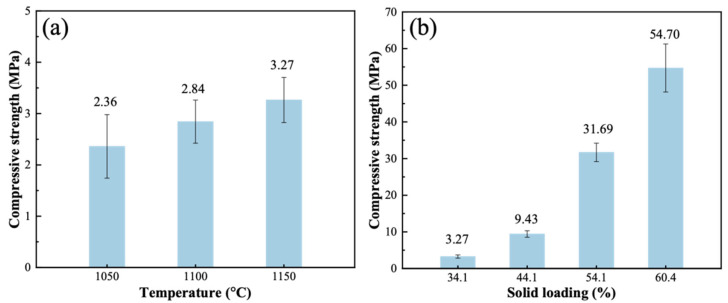
Variation in the compressive strength of the foamed ceramics versus (**a**) sintering temperature at a solid loading of 34.1 wt. % and (**b**) solid loading at a sintering temperature of 1150 °C.

As depicted in Figure 10a, the compressive strength of foamed ceramics exhibits gradual enhancement with increasing sintering temperature. This strengthening phenomenon results from temperature-dependent microstructural evolution. Higher temperatures can promote liquid phase formation and particle sintering, leading to consolidated pore walls and consequently improved mechanical performance of the ceramic structure.

Figure 10b shows the compressive strength of the foam ceramics with different solid loadings. Compressive strength increased significantly with solid loading. The maximum compressive strength (54.7 MPa) was observed, which is attributed to the combination of a 60.4% solid loading and a sintering temperature of 1150 °C. It is known that the compressive strength of porous ceramics is determined by their structure. As mentioned in the previous subsection, with increasing solid loading, the pore size became smaller and the pore walls became thicker, i.e., porous ceramics became denser. This change is the major reason why the compressive strength of ceramics is remarkably improved. It was demonstrated that the change in solid content affects the structure of the ceramics, which in turn affects their compressive strength. Although relatively high compressive strength is an important property of porous ceramics, an increase in solid loading of up to 60.4% leads to a decrease in porosity and an increase in density, which would lead to a degradation of other properties of porous ceramics. Therefore, it is necessary to choose the appropriate solid loading to meet more application scenarios.

### 3.5. Toxicity Leaching Test

The porous ceramics prepared from red mud exhibited both high porosity and strength, making them well-suited for use as insulation materials in the construction industry and as filtration media in environmental treatment applications. Given that these materials may encounter complex environmental conditions, it is critical to assess their toxicity to prevent the leaching of hazardous elements that could threaten environmental and human health. Accordingly, we conducted water and acid leaching tests on the porous ceramic samples, following the Chinese national standard HJ/T299-2007 [40] (Extraction procedure for leaching toxicity of solid waste). The test results are provided in Table 2 and Table 3.

Table 2 displays the concentrations of primary elements in the leachate from red mud after water and acid leaching, measured both before and after sintering at 1100 °C. After sintering at 1100 °C, the concentration of Ca and S in the water leachate increased, while the levels of other elements decreased compared to the raw material’s water leachate. In the acid leachate, the concentrations of Si, S, and P increased after sintering, while the levels of other elements decreased. Table 3 lists the concentrations of hazardous components in the leachate following water and acid leaching, before and after sintering at 1100 °C. To ensure safety, the concentrations of toxic substances leaching from the ceramics must remain below the limits set by the Chinese national standard GB 5085.3-2007 (Table 3) [41,42,43]. All measured concentrations of hazardous metals were significantly below the maximum allowable concentration (MLC) values. These results indicate that porous ceramics derived from red mud can be safely used in applications such as construction and filtration, supporting solid waste recycling without environmental risk.

## 4. Conclusions

This study demonstrated a promising approach to valorizing red mud through the preparation of porous ceramics with uniform pore size distribution and high strength using a foam-gel casting method. The characterization of red mud was conducted to assess its chemical composition, phase composition, morphology, particle size distribution, and liquidus temperature. A systematic examination was conducted to understand the effects of solid content and sintering temperature on the microstructural development of porous ceramics. The results showed that increasing the solid loading reduced porosity and enhanced compressive strength, allowing for the tailoring of mechanical properties to meet specific application requirements. In addition, the increase in solid loading significantly reduced pore size. The densification and strengthening of the ceramic were driven by higher temperatures. This is attributed to enhanced liquid phase formation, while improved particle sinterability is also a key factor. Additionally, toxicity leaching tests confirmed that these ceramics are environmentally safe, with leachate levels well within regulated limits. The findings highlight foam-gelcasting as an effective technique for transforming red mud into a value-added material and reducing environmental hazards associated with red mud accumulation. This approach contributes to sustainable waste management and offers a viable method for the large-scale recycling of red mud into commercially useful porous ceramics, particularly for building insulation and filtration-related environmental applications.

## Figures and Tables

**Table 1:** Chemical composition (wt. %) of red mud as determined with XRF.

Sample	Fe_2_O_3_	Al_2_O_3_	SiO_2_	Na_2_O	TiO_2_	CaO	SO_3_	P_2_O_5_	ZrO_2_
Red mud	36.65	20.44	16.20	8.57	7.10	1.04	0.47	0.26	0.19

**Table 2:** Concentrations of major elements in the leachates of raw red mud and the red mud-based porous ceramic prepared with a solid loading of 34.1 wt. % and sintered at 1100 °C, as determined by water and acid leaching (mg/L).

Procedures	Fe	Al	Si	Na	Ti	Ca	S	P	Zr
Water leaching	16.1	11.4	8.4	403.5	0.72	2.5	6.6	41.9	0.09
Water leaching after sintering at 1100 °C	0.74	5.1	4.4	68.9	0.11	4.5	6.9	5.5	0.009
Acid leaching	8.3	9.7	7.1	271.2	0.43	4.5	9.7	0.53	0.06
Acid leaching after sintering at 1100 °C	1.4	5.5	9.6	85.9	0.28	2.7	24.4	4.0	0.02

**Table 3:** Concentrations of hazardous elements in the leachates of raw red mud and the red mud-based porous ceramic prepared with a solid loading of 34.1 wt. % and sintered at 1100 °C, as determined by water and acid leaching (mg/L).

Procedures	Cu	Pb	Zn	Cr	Cd	Ni	As	Ba
Hazardous component concentration limits	100	5	100	15	1	5	5	100
Water leaching	0.007	0.007	0.002	0.03	0.0004	0.003	0.45	0.01
Water leaching after sintering at 1100 °C	ND	0.004	0.001	0.06	ND	0.0005	0.52	0.002
Acid leaching	0.004	0.008	0.0007	0.02	0.0004	0.002	0.15	0.006
Acid leaching after sintering at 1100 °C	ND	0.004	0.0001	0.08	ND	ND	0.41	0.006

Note: Not detected (ND).

## Data Availability

The original contributions presented in this study are included in the article. Further inquiries can be directed to the corresponding author.
